# Opportunities and Challenges in Twisted Bilayer Graphene: A Review

**DOI:** 10.1007/s40820-020-00464-8

**Published:** 2020-06-13

**Authors:** Amol Nimbalkar, Hyunmin Kim

**Affiliations:** Division of Biotechnology, Daegu Gyeongbuk Institute of Science and Technology (DGIST), Daegu, 42988 Republic of Korea

**Keywords:** Graphene, Twisted bilayer graphene, Magic angle, Superconductivity, van Hove singularities

## Abstract

Two-dimensional (2D) materials exhibit enhanced physical, chemical, electronic, and optical properties when compared to those of bulk materials. Graphene demands significant attention due to its superior physical and electronic characteristics among different types of 2D materials. The bilayer graphene is fabricated by the stacking of the two monolayers of graphene. The twisted bilayer graphene (tBLG) superlattice is formed when these layers are twisted at a small angle. The presence of disorders and interlayer interactions in tBLG enhances several characteristics, including the optical and electrical properties. The studies on twisted bilayer graphene have been exciting and challenging thus far, especially after superconductivity was reported in tBLG at the magic angle. This article reviews the current progress in the fabrication techniques of twisted bilayer graphene and its twisting angle-dependent properties.

## Highlights

- This article presents an overview of twisted bilayer graphene (tBLG) on their fabrication techniques and twisting angle-dependent properties.
- The properties of tBLG can be controlled by controlling the twisting angle between two graphene sheets.

## Graphical abstract

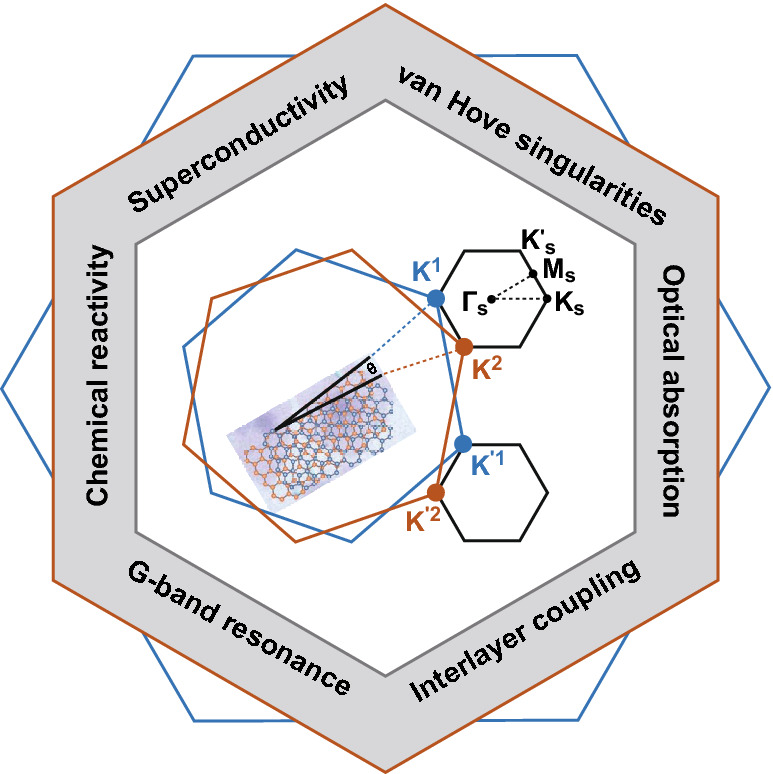

## Introduction

Graphene is composed of a one-atom-thick *sp*^2^ hybridized allotrope of carbon atoms, which takes the form of a two-dimensional (2D) planar honeycomb lattice. It has attracted abundant interest after its first isolation was achieved through the micromechanical cleavage of graphite in 2004 [1–4]. It has been seen as a promising material for applications in sensor, photonic, and electronic devices because of its excellent properties, such as chemical stability, high carrier mobility, low density, and optical transparency [5–9]. However, contrary to the single-layer graphene (SLG), while combining two or more layers of 2D materials in a specific order to fabricate the multilayer structures [10], their mechanical, optical, and electronic properties might be manipulated by varying the stacking order, interlayer spacing, and relative twisting angle [11–16]. The bilayer graphene (BLG) is a simple multilayer structure; in the simple form, two graphene layers ordered in an AB, AA, or a twisted orientation [17–19]. The practical assemblage of distinct graphene layers to make bilayer graphene infrequently leads to an impeccable stacking order; thus, small twisting of a single graphene layer is observed relative to the other [20], which modifies the electronic properties [10, 20, 21]. The atomic orientation among the two layers might further vary, as the bilayer graphene has a weaker interlayer van der Waals bonding due to the lattice deformation, which intensely affects the interlayer electron motion [22–27].

Twisted bilayer graphene (tBLG) is fabricated by the stacking of two monolayers of graphene with a specific twisting angle (*θ*) [28]; in this structure, the moiré pattern has been observed to emerge with a higher periodicity [19, 29]. Recently, the tBLG form of graphene attracted significant attention from several researchers both theoretically as well as experimentally due to its extraordinary optical [30–33] and electronic properties [33–38], which were a result of the development of the moiré patterns. The tBLG systems show the Dirac spectra with twisting angle-dependent (analogous to chirality dependence in carbon nanotube systems) lower-energy van Hove singularities (vHSs), Fermi velocity, magnetoresistance oscillations, and quantum Hall effect (QHE) [6, 17, 18, 21, 39–41]. Therefore, the progress of tBLG-based devices shows excessive potential due to the tunable interlayer coupling and band structure of tBLG [17, 28]. This interest arises predominantly from the physics of tBLG due to the presence of low-lying flat bands near the magic angles [42]. The Fermi energy of the tBLG structure is less than 10 meV near the magic angle (*θ* = 1.05°) [43]; however, upon comparison, the superconductor critical temperature (*T*_c_) ~ 1 K is comparatively high [44]. Meanwhile, several theoretical studies reported the understanding of the superconductor and insulator phases of the tBLG [44–65]. In this review article, we aim to highlight the current progress in the fabrication of tBLG systems as well as their properties and expect to motivate more researchers to work on tBLG systems for different applications.

## Synthesis Methods

**Fig. 1:**
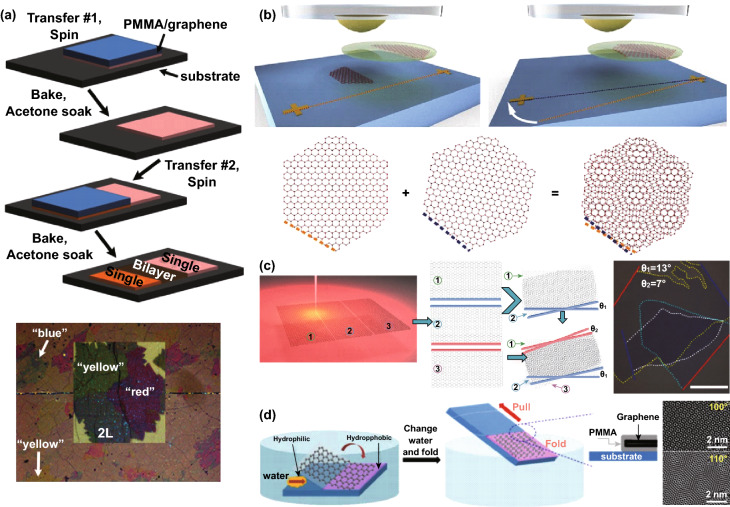
**a** Schematic of the synthesis process flow of twisted bilayer graphene (tBLG) by CVD and optical microscope image of tBLG on SiO_2_/Si substrate [75]. **b** Schematic illustration of detaching an SLG using a hemispherical handle and schematic illustration of the moiré pattern formation [79]. **c** The schematic of the CRS method utilized to fabricate tBLG, double twisted trilayer graphene (DTTG), and optical microscopy image of DTTG [81]. **d** The schematic of the folding procedure of a single-layer graphene sheet with folding driven by delamination only from the hydrophilic surface region, and the removal of polymers and HRTEM images of tBLG on a SiO_2_/Si substrate with twisting angles (100° and 110°) [82]. Adapted with permission from Refs. [75, 79, 81, 82]

In this section, we reviewed some of the recent advanced synthesis methods used to fabricate twisted graphene sheets. The twisted graphene layers are formed on the surface of crystalline graphite naturally due to the accidental folding of graphene layers onto other graphene flakes or themselves [66, 67]. Several techniques have been reported for preparing graphene sheets, such as chemical exfoliation from bulk graphite [68], micromechanical cleavage of highly ordered pyrolytic graphite (HOPG) [69], chemical reduction of chemically exfoliated graphene oxide [70], solid-state graphitization or thermal decomposition of SiC [71, 72], and thermal and plasma-enhanced chemical vapor deposition [73, 74]. Every technique has its own set of advantages and disadvantages. The usually followed method is based on the stacking of two SLGs synthesized by chemical vapor deposition (CVD) to fabricate twisted graphene [75], as shown in Fig. 1a. It usually includes the transfer of CVD-grown single-layer graphene (SLG) onto SiO_2_/Si substrates via a simple wet-chemistry method [76–78], with a protective layer of poly(methyl methacrylate) (PMMA) [78] followed by Cu etching. After the transferring of SLG/PMMA onto the substrate, the PMMA is removed by using acetone and annealing [77]. The twisted bilayer graphene samples are prepared by consecutively transferring a second SLG onto the SLG/SiO_2_/Si in the same way. The prepared sample is baked in the air at 150 °C for 20 min for enhancing the adhesion between the SLG substrate and SLG–SLG [75]. In this technique, the removal of PMMA is a crucial step because if residual PMMA is found at the top and bottom of SLG, it can affect the interlayer interaction among the graphene layers. The control of the twisting angle is difficult in this technique due to the random shapes of SLG grown by CVD.

In another report, Kim et al. [79] reported a new approach by demonstrating the preparation of a small-twist-angle bilayer graphene by utilizing hexagonal boron nitride (hBN) and sequential graphene flake pickup steps by utilizing a hemispherical handle substrate [80], as shown in Fig. 1b. The rotationally aligned transfer process provides a superior control on crystal axes alignment, as well as on small twisting angle due to the controlled flake pickup and selective detachment from the exfoliation substrate. A single graphene flake is divided into two separate regions, as illustrated in Fig. 1b. The divided flakes are then successively picked up by an hBN flake attached to the hemispherical handle. Among the first and the second graphene flake pickup, the substrate is rotated by 0.6° to 1.2° twisting angle with a 0.1° accuracy. The substrate is twisted by a diminutive angle among the two stages. The stem of two graphene flakes from the same graphene layer; a small twist angle is produced among the crystal axes of the distinct layers. The two SLGs are likely to have rotationally aligned crystal axes ensuring an exact tBLG structure because of the graphene single-crystal nature. This fabrication method fulfills the requirements of scientific research and applications due to precise control over the twisting angle. In another report, Chen et al. [81] prepared tBLG by the cutting, rotating, and stacking (CRS) of the graphene layer using femtosecond laser micromachining and a precise transfer method. The single-layer graphene was mechanically exfoliated on a Si/SiO_2_ substrate using a femtosecond laser and was divided into two pieces with a pair of parallel and straight cutting lines, as represented in Fig. 1c. By using the two cutting lines, the two graphene sections were rotated with an angle *θ* and were accurately stacked together onto to Si/SiO_2_ substrate using a suitable transfer method. The control over the twisting angle of tBLG fabricated by this method is better than the other preparation methods.

Wang et al. [82] prepared tBLG films based on the controlled folding of single-layer graphene. There are three steps in this method: (1) the transformation of the SiO_2_/Si substrate to make hydrophilic and hydrophobic sections with a distinct border, (2) the controlled SLG/PMMA delamination from the hydrophilic section in water, and (3) removal of the PMMA layer. The controlled folding in this procedure allows for the fabrication of tBLG structure with distinct stacking orientations; the required twisting angle was achieved by varying the hydrophilic and hydrophobic boundary folding angles. In a recent report, Cao et al. [43] fabricated tBLG by the vertical stacking of graphene to study its superconductivity properties at 1.05° and 1.16° magic angles. In this method, one piece of SLG is fixed, and the other is stacked vertically by the mechanical transfer method on the fixed one at different angles. The vertical stacking method makes it simple and easy to achieve the desired twisting angle in tBLG.

## Lattice and Electronic Structure of tBLG

**Fig. 2:**
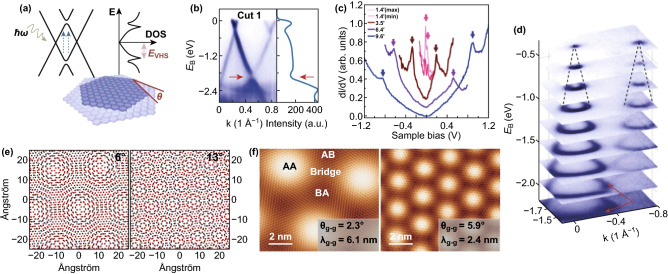
**a** Schematics for band structure with mini-gaps and the corresponding density of state (DOS) with van Hove singularities (vHSs) in tBLG, **b** ARPES spectrum of tBLG [86]. **c** LDOS spectrum of moiré patterns with different twisting angles [98]. **d** Stacking plot of constant-energy contours at different binding energies of tBLG [86]. **e** Moiré patterns of tBLG with 6° and 13° twisting angle [99], **f** STM images of tBLG with 2.3° and 5.9° twisting angle [100]. Adapted with permission from Refs. [86, 98–100]

The variation in the graphene properties is produced due to the mode of stacking orientation among the two graphene layers and the number of stacked graphene layers [83, 84]. The bilayer graphene is fabricated by the vertical stacking of two graphene layers, which results in the breaking of the symmetry of bilayers. The broken symmetry causes inequivalent charge and electrostatic potential among the two graphene layers [85]. However, the tBLG is non-AB-stacked bilayer graphene, in which one graphene sheet rotates by a definite angle (*θ*), as compared to the other [86]. The schematics of band structure and the equivalent density of state (DOS) with van Hove singularities (vHSs) in tBLG are shown in Fig. 2a [86]. The Dirac band dispersions vary drastically and become strongly warped with smaller twisting angles (less than 5°) [87–89]. The Dirac cones of the two distinct layers intersect and produce saddle points in the shared space of tBLG [90], causing the development of van Hove singularities (vHSs) in the density of state (DOS) [91–93], which enhances the Raman *G* band resonance and optical absorption and improves the chemical reactivity of tBLG [93–97]. The band structure of the tBLG domain where the band dispersions are cutting across (Fig. 2b), and the constant-energy contours to the two neighboring Dirac points (Fig. 2d) [86], confirms the formation of the vHSs by the intersection of two Dirac cones. The existence of logarithmic vHSs was also confirmed by Brihuega et al. [98] by using local DOS (LDOS) of two stacked graphene layers with twisting angles between 1° and 10°, as shown in Fig. 2c. The small twist angles in bilayer graphene executed a pattern on the intercalated atoms that causes the modification of the electronic properties (Fig. 2e), which is used as a high-mobility material with significant bandgaps [99]. The moiré patterns are produced through the graphene–graphene interaction by matching the relative orientations of the top-layer graphene lattice, as shown in Fig. 2f. The moiré lattice and strong non-perturbative characteristics of the tBLG in the smaller twisting angle regime are confirmed by Wong et al. [100]. The simplest tBLG also shows unique physical properties because of the controlled interaction among the two Dirac electron gases with a broad moiré pattern [101]. The tBLG with a smaller twisting angle is attended with a moiré pattern that produces particular super-periodicity and uniformity for the electron systems, which causes a substantial variation in the electronic properties [102, 103].

## Raman Spectroscopy

Raman spectroscopy plays a crucial role in the nondestructive analysis of the lattice structure, as well as the optical, electronic, and phonon properties of graphene [104, 105]. Raman spectroscopy is widely utilized to investigate the physical properties of graphene and graphene-based devices [101]. The Raman features dependent on the stacking orientation provide detailed information about the phonon characteristics with their distinctive band structures [106, 107]. The phonon vibration modes of the AB-stacked MLG are separated into an out-plane shear (*C*) vibration modes and in-plane *G* and 2D vibration modes. The first-order Raman endorsed peak in graphene is a *G* band observed at ~ 1584 cm^−1^, accomplishing momentum conservation, which commands that the scattered phonon must carry no momentum. The *D* and *D*′ bands are originated from the intervalley and intravalley double-resonance Raman scattering mechanisms, respectively [108–110]. Two additional *R* and *R*′ modes, which are from the TO and LO phonon branches, respectively [111–113], can differentiate the stacking orientation among the AB stacking and twist. The tBLG superlattice offers a *θ*-dependent *q* wave vector, which triggers phonons inside of the Brillouin zone, due to which the layer breathing vibrations (ZO′ phonons) can be studied in a first-order light scattering [112]. Lui et al. reported that the Raman band is susceptible to interlayer interactions; it can reveal a distinctive line shape for the graphene band of every layer thickness and stacking order [114].

**Fig. 3:**
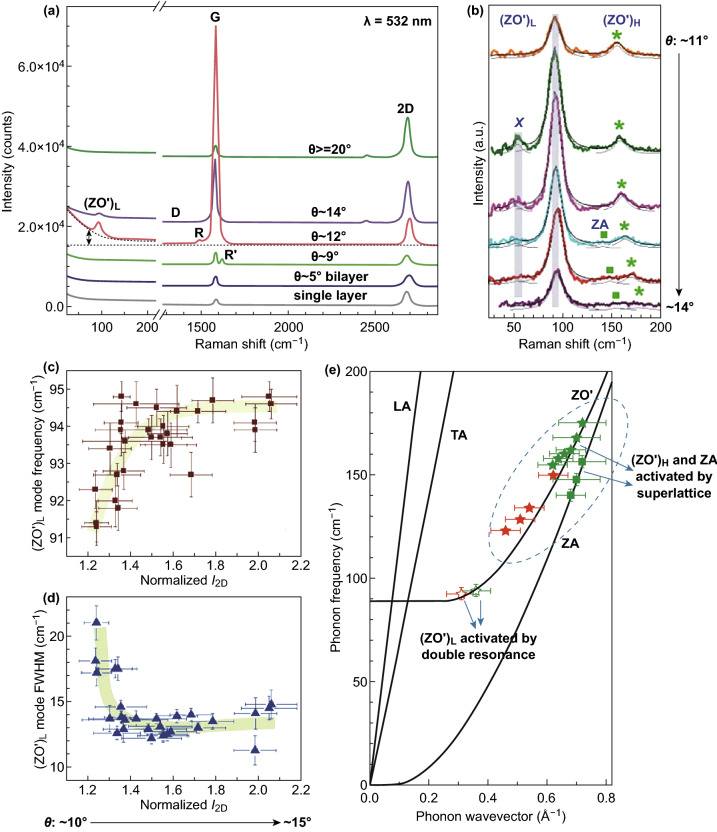
**a** Raman spectrum of tBLG with different twisting angles excited by a 532 nm laser excitation. **b** Background-subtracted low-energy Raman spectrum of six tBLG domains, the gray vertical bars highlight *X* and (ZO′)_*L*_ modes, and the squares and the asterisks highlight ZA and (ZO′)H modes, respectively. **c** Frequency and **d** FWHM of the (ZO′)_*L*_ mode as a function of normalized I_2D_. **e** Low-frequency phonon dispersion [115]. Adapted with permission from Ref. [115]

For investigating the twisting angle effects in tBLG, He et al. [115] carried out the Raman spectroscopy less than 100 cm^−1^ Raman shifts and observed two modes in a smaller range of twist angle. Figure 3 shows the *G* Raman peak intensity is intensely enhanced as a function of twisting angle, which illustrates that these *G* Raman modes and the lower-energy modes share the equivalent resonance amplification mechanism. The intensity of the enhanced *G* peak and the 2D band displays a maximum blueshift than the SLG at 12° twisting angle (called a critical angle) (Fig. 3a). The phonons above 100 cm^−1^ displayed by the Raman scattering process are intermediated by the superlattice wave vector *q*, which simply depends on the twist angle (*θ*) [116, 117]. The background-subtracted lower-energy Raman spectrum for different tBLG domains is shown in Fig. 3b. The observed fundamental layer breathing (ZO′) and out-of-plane acoustic (ZA) modes between 130 and 180 cm^−1^ are activated by the formation of moiré pattern [110, 117]. The additional fundamental layer breathing mode (ZO′)_*L*_ is observed at ~ 94 cm^−1^. The intensities of (ZO′)_*L*_ frequency mode, and the background covering on which the lower-energy Raman lines are comprised, demonstrate substantial resonance enrichment near the 12° twisting angle; it is associated with the *G* Raman peak enhancement. Another Raman mode observed at ~ 52 cm^−1^ is ascribed to the twisting mode due to the rotation between the two graphene layers with respect to each other. The advancement of the frequency and FWHM of the ZO′ mode as a function of *L* normalized *I*_2D_ are shown in Fig. 3c, d. The (ZO′)_*L*_ mode frequency increases with the *I*_2D_ when the twisting angle is less than the critical angle, and the frequency becomes nearly constant, when the twisting angle is greater than the critical angle. This illustrates that the intense variations in the FWHM and frequency of the (ZO′)_*L*_ mode occurred when the twisting angle is close to the critical angle (12°). The low-frequency phonon dispersion shown in Fig. 3e, which shows the phonon frequency observed at ~ 94 cm^−1^, and the phonon wave vector *k*_(ZO′)*L*_ are in good arrangement with the ZO′ phonon dispersion in tBLG and confirming the obligation of this mode with the layer breathing mode (ZO′)_*L*_. The Raman study reveals that the fundamental properties of tBLG are different from the Bernal-stacked bilayer graphene.

**Fig. 4:**
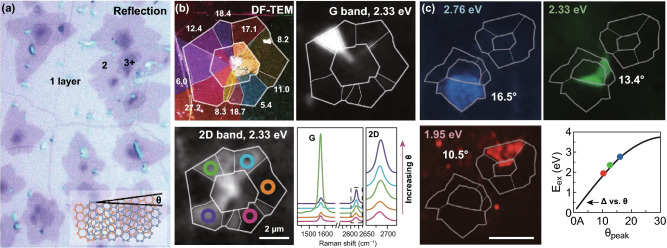
**a** Optical reflection image of CVD-grown graphene transferred on SiO_2_/Si, and inset shows structure of tBLG with a twisting angle *θ*. **b** Dark-field TEM, *G* band, and 2D band Raman images of the same tBLG domain. **c** Wide-field *G* band images of the same tBLG section at three different excitation wavelengths with different *G* band enhancement domains, plot of *E*_ex_ versus *θ*_peak_ [118]. Adapted with permission from Ref. [118]

The Raman imaging reveals the differences in interlayer interactions, which is useful for the investigation of external physical, chemical, and optical properties of bilayer graphene. The white light dissimilarity enhances the reflection image of single and bilayer graphene on SiO_2_ substrate which is shown in Fig. 4a. The bilayer graphene comes out darker than the single-layer graphene with homogeneous intensity. The dark-field TEM image of tBLG domains with different twisting angles is shown in Fig. 4b. The dissimilarities in Raman intensity are associated with the twisting angle, and within the same domain, both bands intensities are nearly constant. The Raman spectra of the same area show the *G* band intensity increases for the ~ 12° twisting angle, attributable to distinctiveness in the joint density of states (JDOS) of tBLG, and its energy is entirely depending on the twisting angle, and the strength of optical transition is directed by the interlayer interactions, which allow direct optical imaging of these parameters, as shown in Fig. 4b. The 2D peak position, intensity, and width vary quickly around the ~ 12° twisting angle, which describes a transition among the low and high twisting angle regions. The wide-field *G* band images of the similar tBLG domain at different excitation energies and the plot of *E*_ex_ versus *θ*_peak_ are shown in Fig. 4d, which reveals the different domains and shows intensity enhancement for different twisting angles corresponding to different excitation energies. The twisting angle with specified excitation energy allows upcoming studies of electrical, mechanical, and optical properties of tBLG at precise angles. In the optical properties of tBLG, parallel band optical transitions play a vital role, which might be further employed in emerging innovative optoelectronic devices with exceptionally tunable features via controlling over the twisting angle [118].

## Optical Properties

**Fig. 5:**
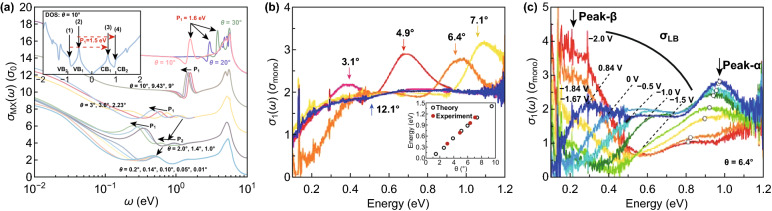
**a** Optical conductivity of some commensurate and incommensurate tBLG structures, and inset shows the leading transition procedures contributing to the formation of the optical conductivity peaks [126]. **b** Optical conductivity *σ*_1_(*ω*) of the different twisting angle tBLG samples, and inset shows comparison of the observed peak position and theoretical prediction. **c** Optical conductivity of gated tBLG (*θ* = 6.4°) for different gate voltages *V*_G_ [127]. Adapted with permission from Refs. [126, 127]

The decreasing in the twisting angle interference among the two lattice periods creates a moiré pattern with a higher wavelength, where the properties of tBLG such as van Hove singularity and bandgaps seem to be present in the far-infrared region and the band velocity of the Dirac cone is considerably decreased [37, 119–122]. The optical absorption study is usually employed for graphene-based systems to examine the electronic structures [122–125]. Recently, Anh Le and Nam Do reported the optical properties of tBLG using a time evolution of states in real space [126]. The optical conductivity of some incommensurate and commensurate tBLG structures is shown in Fig. 5a, which clearly showed the conductivity structure in the infrared-red region. The curves of all groups with different twisting angles reveal that the optical conductivity of tBLG continuously varies with the twisting angle, but the distinctive structure of the twisted systems (*θ* = 0.01° to 0.2°) is found in the lower-energy range. The transition developments for the tBLG structures with *θ* = 10° are shown in the inset of Fig. 5a. The W shape DOS is slowly converted into the U shape by decreasing the twisting angle to zero, which illustrates that the commensurability among the two graphene layers does not play a crucial role in varying the optical and electronic properties. Yu et al. [127] reported the optical absorption spectrum of electrically gated tBLG. The optical conductivity *σ*_1_(*ω*) spectra of tBLG with different twisting angles show two separate interband transitions (Fig. 5b): (1) The frequency-independent conductivity 2*σ*_mono_ comes from the LB transition, and (2) the major absorption peak *α* originates from the transitions vHs_2_ → BE_1_ and BE_2_ → vHs_1_ [128, 129]. Inset of Fig. 5b shows the blueshift of *α*-peak toward the higher energy as the twisting angle increases [129]. The splitting of the absorption edge of *σ*_LB_ into two edges with various energies reveals that the Dirac cones of the bottom and top layers are transferred by varying the total energy [127]. The intensity and energy of peak *α* show variation with the gate bias, as the interlayer potential irregularity breakdowns the configuration of the band edge and vHs. The *V*_G_-driven variation of *σ*_1_(*ω*) for the hole doping region is shown in Fig. 5c. The absorption edge of *σ*_LB_ in Fig. 5c demonstrates a significant expansion, as well as the shifting toward higher energy; it demonstrates the shifting of the *α*-peak toward lower energy, whereas its intensity is decreased noticeably, which confirms the changing of the band structure of tBLG with the gating. The optical absorption spectra showed notable variations like the shifting of inter-van Hove singularity transition peak and the splitting of interlinear band absorption, as well as the appearance of an extremely strong intra-valence band transition.

**Fig. 6:**
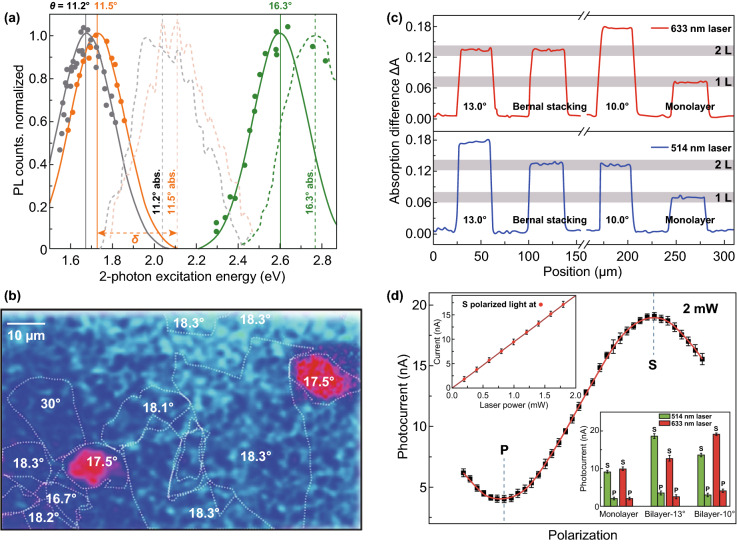
**a** PL excitation peaks tune with *θ *= 11.2°, 11.5°, and 16.3°. **b** Scanning PL map of tBLG collected at 1.26 eV excitation [130]. **c** Line scanning results of the graphene films for different laser energies. **d** The relationship between the incident light polarization and photocurrent and the upper inset image demonstrates the different laser powers and detected photocurrent, the photocurrent of the samples with different twist angles in the lower inset [81]. Adapted with permission from Refs. [81, 130]

The PL excitation spectrum recorded after 2-photon excitation, also the twisting angle-associated tunable linear absorption spectrum, is shown in Fig. 6a [130]. The resonant PL emission was positioned at around ~ 2.0, 2.1 and 2.7 eV, as confirmed by applying 10 nm wide band-pass filters. The domain twisting angles were selected by 1-photon linear absorption spectrum, which spectrally overlaps with the consequent 2-photon PL excitation peaks. The splitting of 2-photon PL excitation and 1-photon absorption peak energies varies with the twisting angle. The PL map of tBLG structure collected at around 1.26 eV excitation shows a significant PL emission enhancement after the 2-photon excitation of 17.5° domains, as compared to the nearby domains (Fig. 6b) [130]. The appreciative band-pass optical filtering confirms the emission energy matches well with the 1-photon absorption resonance at ~ 2.8 eV of the 17.5° domain. This resonant PL variation with the vHs reveals that the electrons will thermalize quickly to low metallic continuum states by electron–electron scattering. The line scanning results of the different graphene films by two different lasers wavelength (633 and 514 nm) are shown in Fig. 6c [81]. The signal is increased by ~ 35% for a 13° twisting angle tBLG domain with 514 nm laser wavelength, and a 10° twisting angle tBLG domain shows similar results for a 633 nm laser wavelength. The photocurrent of tBLG films might be extensively enhanced by both *s*- and *p*-polarized lights with consequent laser wavelength consistent with the absorption enhancement, as shown in Fig. 6d [81]. The photocurrent and optical absorption enhancement of tBLG could be achieved by varying the twisting angles.

**Fig. 7:**
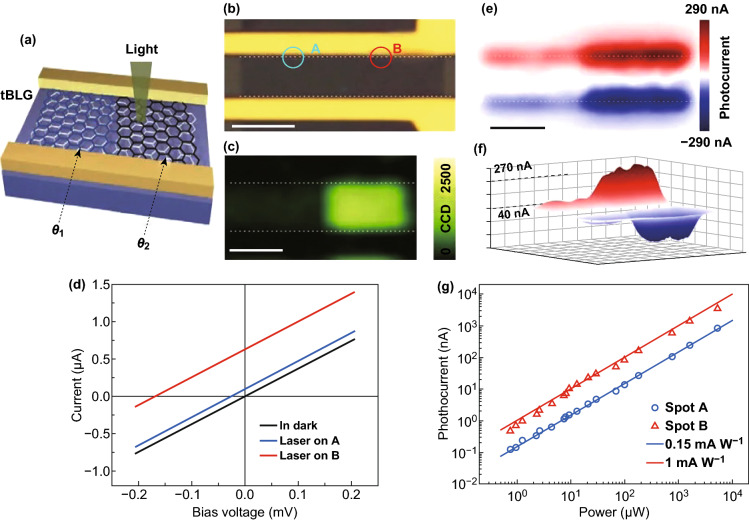
**a** Schematic representation of a tBLG photodetection device, which comprises of two adjacent tBLG domains with different twisting angles of 7° and 13°. **b** Optical image of the tBLG photodetection device. **c** Raman *G* band intensity mapping image of 13° twisting angled tBLG with an enhanced *G* band intensity at 2.33 eV laser energy. **d** Current versus source–drain bias curve with laser focus on spot A of 7° and spot B of 13° twisting angle in tBLG with laser off. **e**, **f** Scanning photocurrent image and its 3D view of the tBLG device. **g** Photocurrents generated as a function of incident power at spot A of 7° and spot B of 13° twisting angle of the tBLG device [86]. Adapted with permission from Ref. [86]

Recently, Yin et al. [86] reported a strong light-matter interaction and selectively improved photocurrent generation of tBLG devices under laser illumination for different twisting angles. The schematics of two parallel tBLG films with 13° and 7° twisting angle on a SiO_2_/Si substrate embedded with two terminals are shown in Fig. 7a, b. The intensity of *G* band of 13° twisted tBLG domain showed a consistent 20-fold enrichment compared with the 7° twisted tBLG domain, as shown in Fig. 7c. The interfacial junctions of tBLG metal electrodes were utilized to separate the photo-excited electrons and holes on the tBLG domain under the laser light [131, 132]. The 13° and 7° twisted tBLG domains produce distinct photocurrent shifts, as shown in Fig. 7d. The 13° and 7° twisted tBLG domains produce 0.63 and 0.097 mA net photocurrent, respectively, at zero bias with a 532 nm laser. The net photocurrent mapping of the tBLG devices shows converse directions at two graphene electrode interfaces; for example, the intensity of photocurrent of 13° twisted tBLG domain is ~ 6.6 times greater than the 7° twisted tBLG domain (Fig. 7e, f). The photocurrent from both 13° and 7° twisted tBLG domains increases as the incident 532 nm laser power increases from ~ 1 μW to ~ 5 mW, as shown in Fig. 7g. The substantial enhancement in photoresponsivity of 13° twisted tBLG domain below the enlightenment of different incident 532 nm laser power is observed. This twisting angle-dependent photocurrent enhancement holds enormous promise for high-selectivity photodetection applications.

## Electronic Properties

**Fig. 8:**
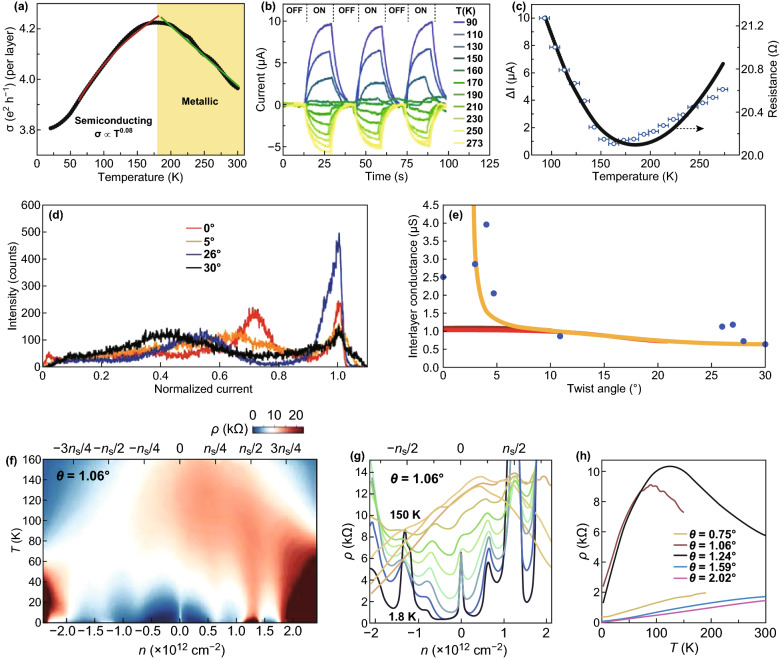
**a** Variation in temperature-dependent conductivity, *σ*, of the tMLG device at 90 K to 273 K temperature range, and blue and red lines are power law and linear fit to the curves in the metallic region and semiconducting region, respectively. **b** Temporal variation in the photocurrent with the intermittent enlightenment of light source measured at 50 mV. **c** Photoresponse variation indicated by the varying of the current (left) and the resistance (right), measured at different temperatures [134]. **d** Normalized current distributions of four tBLG domains with different twisting angles. **e** Interlayer conductance of tBLG with different twisting angles. The orange curve and the red curve indicate a phonon-assisted transport mechanism with and without considering the renormalization of the Fermi velocity, respectively [135]. **f** Temperature-dependent resistivity (*ρ*) of tBLG device. **g** Resistivity (*ρ*) as a function of carrier density at selected temperatures in tBLG device, **h** *ρ*(*T*) recorded in tBLG devices with different twisting angles near − *n*_s_/2 filling [137]. Adapted with permission from Refs. [134, 135, 137]

The perfect and superior characteristics of the bilayer graphene in twisted multilayer graphene (tMLG) than the suspended form of graphene could be attributed to the fact that the tMLG is tens of nanometer in thickness and maintains the graphene layers ultra-clean, as well as free from any substrate influence. These extraordinary properties in tMLG generate from the higher degree of decoupling that occurs from the angular twisting between the layers [133]. Recently, Mogera et al. [134] reported the semiconducting to metallic transition converging behavior of twisted multilayer graphene (tMLG). The temperature-dependent conductivity (*σ*) of the tMLG device in the 90 K to 273 K temperature range is shown in Fig. 8a. As the temperature increases, the conductivity per layer in the tMLG slowly increases and reaches a maximum at around 180 K and then linearly decreases up to 300 K, which reveals the variation in non-monotonous conductivity with a distinctive semiconducting to metallic conversion on raising the temperature. The sequential difference in device photocurrent under light exposure is shown in Fig. 8b. The photocurrent increases in the semiconducting region and drops with the rising temperature in the metallic region and decreases with no photoresponse at a transition temperature (Fig. 8c). These pristine properties reveal the decoupled nature of the graphene layers in tMLG.

The interlayer contact conductance among the BLG with different twisting and stacking structures synthesized by the CVD method is recently discussed by Yu et al. [135]. A statistical method is applied for comparing the twisting angle-dependent current in the tBLG domains. The statistical results for different tBLG domain with various twisting angles are shown in Fig. 8d. The tBLG with a small twisting angle displays a higher current, which indicates excellent contact conductance at no twisting among the graphene layers. Figure 8e shows the interlayer conductance of tBLG with various twisting angles. The interlayer contact conductance decreases with an increase in the twisting angle. The twisting angle propagation to the interlayer potential energy enhances, at the larger twisting angle [136]. The interlayer contact conductance of 0° BLG domain is ~ 4 times higher than the 30° tBLG domain, which reveals the twisting angle-dependent graphene interlayer contact conductance originated from the decoupling and coupling transitions.

Polshyn et al. [137] discussed the electrical transport measurements for different tBLG devices with 0.75° to 2° twisting angles in the room temperature. The resistivity (*ρ*) of the tBLG domain (*θ* = 1.06°) measured near the flat band condition for carrier densities spanning the lower-energy band is shown in Fig. 8f, g. The resistance peaks or insulating phases at some integer multiples of *n*_s_/4 and superconducting states at different partial band fillings are revealed in Fig. 8f. At nearly all densities, the resistivity (*ρ*(*T*)) increases with the increase in temperature and remains steady with the metallic behavior. The resistivity (*ρ*(*T*)) measured in tBLG devices with different twisting angles near − *n*_s_/2 is shown in Fig. 8h. The resistivity is enhanced sub-linearly with the increase in temperature and reaches the highest point at a temperature *T*_H_; the resistivity scales linearly with temperature below the temperature *T*_H_. At the lowest temperatures, resistivity diverges from a linear dependence on temperature. The tBLG devices clearly show the resistivity saturation, superconducting, or insulating behavior at moderate temperature regimes. The observed three distinctive temperature regimes are noticeable by the different behavior of resistivity (*ρ*(*T*)) depending on the carrier density and twisting angle.

## Superconductivity

**Fig. 9:**
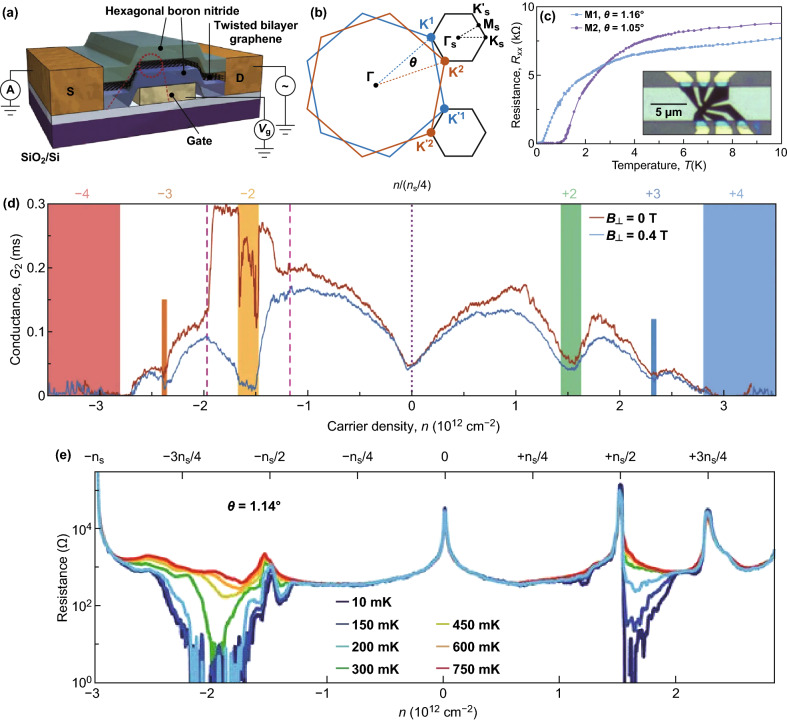
**a** Schematic of the tBLG and hexagonal boron nitride-sandwiched device. **b** The mini-Brillouin zone is made from the difference between the two *K* (or *K*′) wave vectors for the two graphene layers [43]. **c** Four-probe resistance *R*_xx_ determined in two devices *M*1 and *M*2 with twisting angle of 1.16° and 1.05°, respectively, and the optical image of the device is shown in inset where the darker region is the patterned tBLG. **d** Two-probe conductance (*G*_2_ = *I*/*V*_bias_) of the device M1 calculated in zero magnetic field (red) and at a vertical field of *B*_⊥_ = 0.4 T (blue) [141]. **e** Temperature dependence of the resistance over the density range required to fill the moiré unit cell [143]. Adapted with permission from Refs. [43, 141, 143]. (Color figure online)

The unusual superconducting behavior of different materials has been studied broadly for the last decades. The weak interlayer interaction creates the interlayer coupling in tBLG, and the strength of interlayer coupling as well as twisting angles equally affects the Fermi velocity and the VHSs of tBLG, which makes the novel electronic state of tBLG, which is different from those in SLG [138–140]. The twisting angle among the layers of bilayer graphene decides the degree of interlayer coupling and plays a decisive role in its electronic properties [140]. Recently, Cao et al. [141] reported the unconventional superconductivity in the magic-angle graphene superlattices. The representative device structure of the encapsulated tBLG is shown in Fig. 9a. The mini-Brillouin zone is built from the variation present among the two *K* or *K*′ wave vectors for the two graphene monolayers (Fig. 9b). The interlayer hybridization takes place between the Dirac cones in each valley, where the intervalley interactions are intensely suppressed [120, 142]. The longitudinal resistance (*R*_xx_), as a function of temperature (*T*(K)) for two tBLG devices with 1.16° and 1.05° twisting angles, demonstrated zero resistance at 70 mK revealing the superconducting state (Fig. 9c). The critical temperature (*T*_c_) determined using a resistance of 50% of the non-superconducting state value was about 1.7 and 0.5 K for the two tBLG devices. The two-probe conductance versus carrier density at zero magnetic fields and a 0.4 T perpendicular magnetic field of *M*1 device is shown in Fig. 9d, which clearly shows the V-shaped conductance created from the renormalized Dirac cones of the tBLG band structure at charge neutrality point (*n* = 0). It also shows the states of insulating at the superlattice bandgaps *n *= ± *n*_s_. The insulating state at ± 3.2 × 10^12^ cm^−2^ is as a result of the presence of single-particle bandgaps in a band structure, as well as the observed conductance minima connected with many body gaps [43]. At 70 mK temperature and − 1.3 × 10^12^ to − 1.9 × 10^12^ cm^−2^ electrons per unit cell, the conductance was significantly high for nil magnetic fields than in the vertical 0.4 T magnetic field (*B*_⊥_), which reveals the presence of superconductivity at the magic angle. The density-dependent resistance of the tBLG device with a 1.14° twisting angle plotted almost over the complete flat band density range is shown in Fig. 9e [143]. The tBLG device showed lower charge carrier inhomogeneity (*δn* < 2 × 10^10^ cm^−2^), and at the magic angle (*θ* = ~ 1.1), the resultant hybridization and moiré superlattice among the graphene layers caused the development of a remote flat band at the charge neutrality point (CNP) [120, 143]. In the flat band, the resistive states are observed at the charge neutrality point (CNP) and ± *n*_s_/2 and +3 *n*_s_/4. The superconductivity regions emerge in both electron- and hole-doped regions at ~ 10 mK base temperature with the dropping of resistance to zero. However, for ± *n*_s_/2 densities, below the base temperature, no sign of superconductivity is observed. The superconductivity appears considerably absent or weak on the lower-density side and extra stronger in the higher-density side of the insulator in both bands.

**Fig. 10:**
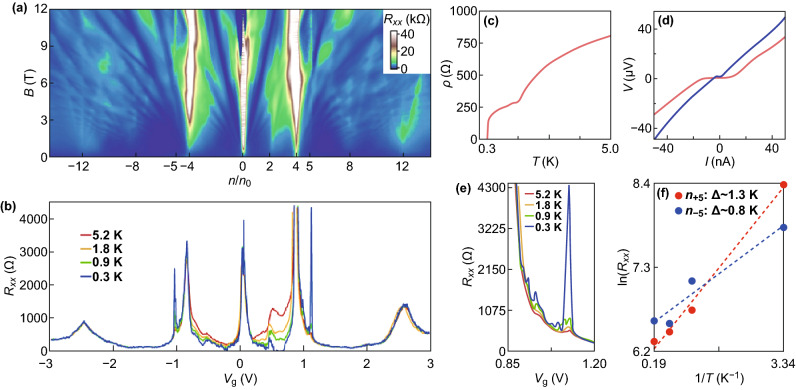
**a** *R*_xx_ versus magnetic field *B* and gate voltage *V*_g_, showing a Landau fan pattern. **b** *R*_xx_(*V*_g_) at different temperatures. **c** *ρ* versus temperature when the density is tuned to the superconducting phase. **d** Voltage–current characteristics at *T* = 280 mK and *V*_g_ = 0.58 V (red) and 0.50 V (blue). **e** Temperature dependence of the resistance peak. **f** Arrhenius plot of resistance [144]. Adapted with permission from Ref. [144]. (Color figure online)

Recently, Codecido et al. [144] reported both insulating state and superconductivity in a tBLG structure at a ~ 0.93° twisting angle. The ~ 0.93° twisting angle is 15% small than the previously reported magic angle (*θ* = 1.1 ± 0.1°) [43, 139, 140]. The magnetic field (*B*) and longitudinal resistance (*R*_xx_) versus an elongated gate voltage (*V*_g_) range at a 1.7 K temperature (Landau fan pattern) are shown in Fig. 10a. The satellite peak is observed at *V*_g_ = ± 0.85 V, as the lower-energy moiré bands are filling at densities *n*_m_ = ± 4 (where *n*_m_ is the number of charges per moiré unit cell) [43, 141]. The resistance peak appears at *V*_g_ = 0.43 V from which an alongside set of Landau levels emanate. The peak on the half-filling and the twofold degeneracy of Landau levels illustrate the breaking of the symmetry of spin valley [145] and the development of a novel quasi-particle Fermi surface. At *n*_m_ = 0, ± 4, and +2, the resistance peaks are clearly observed in *R*_xx_(*V*_g_) at *B* = 0 and for different temperatures (0.28 to 5.2 K) (Fig. 10b). The *R*_xx_ is zero at *T* = 280 mK, for 0.51 < *V*_g_ < 0.65 revealing the development of superconductivity [43]. The development of superconductivity and conceivably penetrating superconducting regions might be responsible for the increase in *R*_xx_ with the increase in temperature at *n*_m_ = 2. As the temperature (*T*) decreases, *ρ* decreases to zero with the two consecutive steep successions at *T* ~ 0.3 K and *T* ~ 1.5 K (Fig. 10c); it could be associated with non-Planckian dissipation of the extraordinary metal states [146]. The voltage–current (*V*–*I*) curves at two descriptive densities (*V*_g_ = 0.58 V and *V*_g_ = 0.50 V) are shown in Fig. 10d. The maximum value of the critical current (*I*_c_) is observed at *V*_g_ = 0.58 V and observed the supercurrent for an elongated range of density with *I*_c_ (~ 1 to 15 nA). The temperature dependence of the resistance peaks is nearly invisible, as the temperature is enhanced above ~ 5 K (Fig. 10d). The Arrhenius plot of resistance with ~ 1 K energy gap is shown in Fig. 10f. The peaks at *n*_m_ ≈ ± 5 are unlikely to initiate from the angular disorder and single-particle gap as a result of an alignment between graphene and hBN [147, 148]; these features are uncertainly attributed to the development of a novel interrelated insulating state. *θ* = 0.93° is the lowest twisting angle reported till to the date for tBLG devices showing superconductivity and insulating state.

## Conclusions

The objective of this review paper is to provide detailed information regarding the fabrication of twisted bilayer graphene (tBLG) via different methods, its properties, as well as its technological applicability. The tBLG-related research field has developed at an enormous speed. The prominent tBLG fabrication methods such as micromechanical exfoliation, CVD, graphene flake pickup, CRS, stacking methods, and their unique properties are summarized in the initial sections. The control over the twisting of two graphene layers is the major challenge in the fabrication of tBLG. The twisted bilayer graphene (tBLG) is a novel arrangement, which shows the basic properties are different from those of the stacked bilayer graphene. The variation in line width and position of the (ZO′)_*L*_ mode illustrates the influence of the twisting angle-dependent electronic band overlaps, onto the Raman spectrum. The continuous variation in optical and electrical properties of tBLG is strongly dependent on the twisting angle (*θ*) among the two graphene layers. We believe that the development and variation in the optical properties of tBLG would be extensively used in the future in the field of optoelectronics. The tBLG devices displayed non-monotonous conductivity variation, which reveals a semiconductor to metallic transition. The superconducting properties observed in tBLG are due to the electron interactions, which can distinctly influence the properties of moiré superlattices at higher densities and smaller twisting angles. There are quite a few challenges that are related to achieving a control over the twisting of two graphene layers for the development in the fabrication and characterization of twisted bilayer graphene (tBLG). It is expected that theoretical studies will be published in the future to search for novel superconducting and insulating phases of tBLG at a lower temperature.
